# Contrast Agents in Breast MRI: State of the Art and Future Perspectives

**DOI:** 10.3390/biomedicines13040829

**Published:** 2025-03-31

**Authors:** Filippo Pesapane, Adriana Sorce, Ottavia Battaglia, Carmen Mallardi, Luca Nicosia, Luciano Mariano, Anna Rotili, Valeria Dominelli, Silvia Penco, Francesca Priolo, Gianpaolo Carrafiello, Enrico Cassano

**Affiliations:** 1 Breast Imaging Division, IEO European Institute of Oncology IRCCS, 20141 Milan, Italy; ottavia.battaglia@ieo.it (O.B.); carmen.mallardi@unimi.it (C.M.); luca.nicosia@ieo.it (L.N.); anna.rotili@ieo.it (A.R.); valeria.dominelli@ieo.it (V.D.); silvia.penco@ieo.it (S.P.); francesca.priolo@ieo.it (F.P.); enrico.cassano@ieo.it (E.C.); 2 Postgraduation School in Radiodiagnostics, Università degli Studi di Milano, 20122 Milan, Italy; gianpaolo.carrafiello@unimi.it; 3 Department of Oncology and Hemato-Oncology, University of Milan, 20122 Milan, Italy; 4 Foundation IRCCS Cà Granda-Ospedale Maggiore Policlinico, 20122 Milan, Italy

**Keywords:** breast MRI, gadolinium-based contrast agents (GBCAs), tumor neoangiogenesis, abbreviated breast MRI (AB-MRI), radiology, breast cancer

## Abstract

Contrast-enhanced magnetic resonance imaging (CE-MRI) has become an essential modality in breast cancer diagnosis and management. It is particularly used for locoregional staging, high-risk screening, monitoring treatment response, and assessing complications related to breast implants. The integration of gadolinium-based contrast agents (GBCAs) enhances the sensitivity and specificity of CE-MRI by providing detailed morphological and functional insights, particularly highlighting tumor neoangiogenesis. Despite its advantages, CE-MRI faces challenges such as high costs, limited accessibility, and concerns about gadolinium retention in tissues, prompting ongoing research into safer, high-relaxivity contrast agents like gadopiclenol. Advances in multiparametric imaging, including dynamic contrast-enhanced sequences and diffusion-weighted imaging, have refined diagnostic accuracy, enabling precise staging, and treatment planning. The introduction of abbreviated breast MRI (AB-MRI) protocols offers a promising solution to barriers of cost and scan duration, maintaining diagnostic efficacy while improving patient accessibility and comfort. Future innovations in contrast agents, imaging protocols, and patient-centered approaches hold the potential to further enhance the utility of breast MRI, ensuring equitable and effective application in global healthcare systems.

## 1. Introduction

Contrast-enhanced breast magnetic resonance imaging (CE-MRI) has established itself as a valuable imaging technique in breast cancer diagnosis and management. Its primary applications include locoregional staging of known breast cancer, screening of high-risk women, monitoring of treatment response, and assessing implant-related complications. CE-MRI is particularly valuable in detecting multifocal, multicentric, and contralateral disease, which can significantly impact surgical planning and therapeutic decision-making [1,2,3].

Gadolinium-based contrast agents (GBCAs) enhance MRI’s sensitivity and specificity by highlighting tumor-associated neoangiogenesis, a hallmark of malignancy. The dynamic contrast-enhanced (DCE) MRI approach allows for characterization of lesion enhancement patterns, facilitating differentiation between benign and malignant findings. In addition to cancer detection, CE-MRI is widely used for evaluating tumor response to neoadjuvant chemotherapy (NAC), providing essential information on residual disease and treatment efficacy [4,5]. CE-MRI also plays a pivotal role in screening women at high genetic or familial risk of breast cancer, such as those with BRCA1/2 mutations, where its superior sensitivity compared to mammography and ultrasound improves early detection. Additionally, it is increasingly considered for women with dense breast tissue, where conventional imaging methods may have limited diagnostic performance. The emergence of abbreviated breast MRI (AB-MRI) protocols has made this technology more accessible by reducing scan times and costs while maintaining high diagnostic accuracy [4,5,6].

Despite the significant benefits of CE-MRI, concerns regarding the safety and long-term retention of GBCAs have led to ongoing research into safer, more stable high-relaxivity contrast agents. The development of novel macrocyclic GBCAs, such as gadopiclenol, aims to optimize contrast enhancement while minimizing potential risks. As imaging technology advances, continued refinement of contrast agents and MRI protocols will further enhance the role of CE-MRI in personalized breast cancer care [6]. This review aims to provide an overview of the current state of the art in breast MRI, with a particular focus on contrast agents, their clinical indications, and future directions for advancing breast MRI as a diagnostic and screening tool.

## 2. State of the Art of Contrast-Enhanced Breast MRI

### 2.1. Multiparametric Breast MRI Protocols

Breast MRI has evolved from being primarily a contrast-enhanced imaging modality to a multiparametric approach, routinely incorporating T2-weighted and diffusion-weighted imaging (DWI). Breast lesions may appear with heterogeneous enhancement or as ill-defined enhanced areas. To address these challenges, dynamic contrast-enhanced T1-weighted sequences are crucial for differentiating benign from malignant lesions. A native T1-weighted image must be obtained before contrast administration.

According to the EUSOMA working group, for breast MRI, it is recommended to use two-compartment (vascular/interstitial) gadolinium-chelates at a standard dose of 0.1 mmol/kg with an injection rate of 2–3 mL/s, followed by saline flushing (20–30 mL at 2 mL/s) [7].

The section thickness of T1-weighted acquisitions should not exceed 2.5 mm to ensure detection of enhancing cancers measuring 5 mm or larger [1]. It is critical to acquire images 60–70 s post-contrast injection to classify the initial enhancement phase (slow, medium, or rapid), as malignant lesions typically exhibit peak enhancement at this time [8].

Dynamic analysis of delayed enhancement involves assessing signal intensity changes over time by acquiring a series of T1-weighted images between 5 and 7 min after contrast administration. Delayed enhancement is categorized into persistent (increasing), plateau (stable), or washout (decreasing) patterns [8].

T2-weighted imaging with and without fat suppression should be acquired as most malignant breast lesions usually show a significant signal intensity loss in T2-weighted images compared to normal breast tissue because of the high cellularity and low water content [9].

DWI is performed by applying magnetic field gradient pulses (b-factors) to a T2-weighted echo-planar imaging sequence. The apparent diffusion coefficient (ADC) quantifies water diffusivity, which is inversely correlated with tissue cellularity and membrane integrity [10].

Malignant lesions generally have lower ADC values due to restricted water diffusion caused by their dense cell structure, aiding in the differentiation from benign or cystic lesions.

### 2.2. Breast MRI Indications

Breast MRI has several clinical indications [Table 1]; it is particularly useful for high-risk screening of women with a high lifetime risk of developing breast cancer (genetic mutations such as BRCA1, BRCA2, PTEN, P53, STK11, and CDH11 and strong family history) [11].

These patients face not only a high risk of developing breast cancer but are also more likely to develop aggressive histotypes at a younger age compared to the general population. Contrast-enhanced MRI represents a powerful diagnostic tool in addition and a potential solution to concerns about radiation dose associated with conventional mammography in young BRCA-mutated women [12].

MRI also plays a role in treatment planning and evaluating treatment response of NAC or other treatments, characterizing equivocal findings from conventional imaging, and evaluating patients with suspected occult primary breast cancer or inflammatory breast cancer [11].

Other specific applications include guiding biopsies when lesions are only visible on MRI, evaluating patients with breast discharges or with suspected breast cancer, and staging DCIS [11].

In particular, contrast-enhanced breast MRI has become a key tool to accurately study patients newly diagnosed with an invasive lobular cancer. Indeed, due to an insidious proliferative pattern with a lack of desmoplastic and fibrotic reactions [13], in many cases lobular carcinoma remains clinically and radiologically elusive, thus representing a diagnostic challenge. In this scenario, a much higher sensitivity of contrast-enhanced MRI for ILC detection has been demonstrated compared to conventional breast imaging modalities such as ultrasound and mammography, with sensitivity rates for MRI ranging from 83% to 100% [14,15].

**Table 1:** Breast imaging indications across EUSOBI [16], the European Commission [7,17], the United States [18], the United Kingdom [19], and Canada [20].

Indication	EUSOBI	European Commission	United States	United Kingdom	Canada
Screening for high-risk populations	YES	YES	YES	YES	YES
Breast implants (implant integrity, silicone leakage)	YES	YES	YES	Not specified	Not specified
Assessment of newly diagnosed breast cancer	YES	YES	YES	YES	YES
Dense breasts	Not specified	Not specified	Not specified	YES	Not specified
Response to NAC	YES	Not specified	Not specified	YES	YES
Suspected recurrent or residual disease	Not specified	YES	YES	Not specified	YES
Assessment of inconclusive findings on other imaging	Not specified	YES	Not specified	Not specified	Not specified
Guidance for interventional procedure	Not specified	YES (biopsy, localization)	Not specified	Not specified	Not specified
Suspicious occult lesions on imaging	YES	YES	Not specified	Not specified	Not specified

## 3. Gadolinium Breast MRI Contrast Agents

Gadolinium-based contrast agents (GBCAs) are pharmaceutical products capable of selectively increasing the SI of blood or of tissues in which they are localized by influencing the signal-producing properties of hydrogen nuclei [21].

This occurs by altering the signal-producing properties of hydrogen nuclei, leading to a reduction in relaxation times. This effect is most pronounced in T1-weighted imaging, where it produces a noticeable contrast enhancement, resulting in increased brightness of the targeted structures relative to adjacent tissues [21].

Consequently, GBCAs facilitate more precise identification of enhanced structures, thereby improving diagnostic sensitivity and specificity [21].

As implied by their name, the main character of GBCAs is the highly paramagnetic metal gadolinium (Gd^3+^), a trivalent lanthanide ion with seven unpaired electrons [22].

Gd^3+^, per se, is considered a toxic ion as, having a similar ionic radius, it can compete with Ca^2+^, binding with higher affinity to the Ca^2+^-binding enzyme and altering the kinetics of the biological processes catalyzed in all the systems that require calcium for proper functioning, such as neurotransmission and muscle contraction, among others [22].

For this reason, in clinically approved MRI contrast agents, gadolinium is chelated by an organic ligand, rendering it unavailable for interaction and thus minimizing its toxicity, facilitating elimination, and reducing biotransformation and accumulation [23].

**Figure 1:**
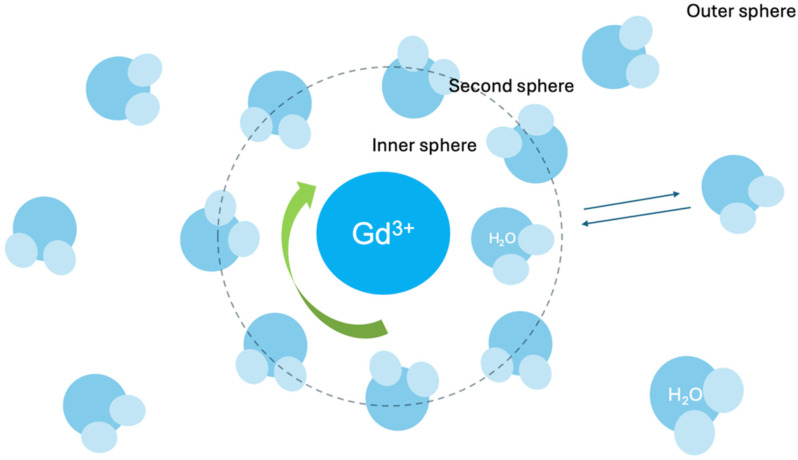
Gadolinium-inner sphere.

In aqueous solution, a free, unchelated gadolinium ion (Gd^3+^) exists surrounded by a hydration sphere of approximately eight or nine water molecules, and these water molecules are in the inner coordination sphere, directly bound to the Gd^3+^ ion (Figure 1) [23].

Each water molecule is oriented so that the oxygen atom (the more electronegative part of the water molecule) is pointing towards the Gd^3+^ ion and it happens because Gd^3+^ ion has a positive charge while the oxygen atom of water carries a partial negative charge. These water molecules directly bound to the Gd^3+^ make up the inner coordination sphere.

GBCAs utilize chelating agents (ligands) that are organic molecules designed to bind tightly to the Gd^3+^ ion. A typical ligand offers multiple coordinating protons (often oxygen or nitrogen) that displace most of the inner-sphere water molecules initially surrounding the free Gd^3+^ and capturing it within the chelate’s structure [23].

Despite chelation, one water molecule typically remains in the inner coordination sphere of Gd^3+^ ion (inner-sphere water molecule), and is essential for the contrast-enhancing effect [23].

The single inner-sphere water molecule rapidly exchanges with water molecules from the surrounding tissue (called the “bulk water” of the tissue) [23].

The crucial process creating the contrast enhancement effect is the rapid exchange between the inner-sphere water molecule and the bulk water molecules; the inner-sphere water molecule constantly detaches from the Gd^3+^ and is replaced by a bulk water molecule, which then becomes the new inner-sphere water. It happens extremely quickly, i.e., a million times per second [23].

Each time a water molecule briefly interacts with the Gd^3+^, it experiences the strong magnetic field of the gadolinium. This interaction alters the relaxation of water molecules [23].

Each contrast agent, according to the specific physical and chemical properties, has a different water exchanging rate that, in turn, is going to influence the relaxation efficiency of a given GBCA [23].

The more GBCA that is present, the more pronounced this effect, and the brighter the signal in that area on an MRI image.

GBCAs can be categorized according to either the chemical structure of the ligand (macrocyclic or linear) or the electric charge of the GBCA (ionic or non-ionic) into four main categories [24] [Table 2]:

- Linear ionic:
  ⚬ Gd-DTPA, gadopentetate dimeglumine (Magnevist);
  ⚬ Gd-BOPTA, gadobenate dimeglumine (MultiHance);
  ⚬ Gd-EOB-DTPA, gadoxetate disodium (Eovist, Primovist);
  ⚬ MS325, gadofosveset trisodium (Vasovist, Ablavar).
- Linear non-ionic:
  ⚬ Gd-DTPA-BMA, gadodiamide (Omniscan);
  ⚬ Gd-DTPA-BMEA, gadoversetamide (OptiMARK).
- Macrocyclic ionic:
  ⚬ Gd-DOTA, gadoterate meglumine (Dotarem, Artirem).
- Macrocyclic non-ionic:
  ⚬ Gd-HP-DO3A, gadoteridol (ProHance);
  ⚬ Gd-BT-DO3A, gadobutrol (Gadovist, Gadavist);
  ⚬ Gadopiclenol (Elucirem, Vueway).

Linear GBCAs have an open-chain chelate structure that is less stable than the macrocyclic structure and therefore is inherently more susceptible to releasing free gadolinium ions in vivo, especially in the presence of endogenous ions like zinc or copper, or under conditions of low pH [23].

Macrocyclic GBCAs feature a closed, ring-like chelate structure that more effectively cages gadolinium ions. The ring typically involves nitrogen protons, along with other coordinating nuclei like oxygen, contributing to the cage-like structure. This macrocyclic structure makes them more stable and less likely to release free gadolinium [23].

The chelating ligands influence the key properties of the GBCA, particularly their stability; in ionic linear GBCAs, electrostatic interactions between the positively charged gadolinium ion (Gd^3+^) and the negatively charged carboxylate groups within the chelating ligand are stronger compared to non-ionic linear agents [23]. However, linear GBCAs, both ionic and non-ionic, are still less stable compared to macrocyclic GBCAs due to the open-chain structure of linear agents, which leaves the Gd^3+^ more exposed and susceptible to transmetallation or other reactions that lead to the release of free gadolinium [23].

All GBCAs, independently of the type, ultimately shorten both the longitudinal (T1) and transverse (T2 and T2*) relaxation times of water protons in tissues. This effect is the basis of their contrast-enhancing properties in MRI [23].

In the past, Mn^2+^ and Fe^3+^ were investigated and Gd^3^-based contrast agents were explored to assess the suitability of these ions as contrast agents and, among them, gadolinium showed the most promising enhancing effect [23,25].

Typically used in the +2 oxidation state (Mn^2+^), manganese dipyridoxyl diphosphate was the first manganese-based contrast agent introduced for human use in 1997 as an intravenous hepatobiliary MR, but concerns about toxicity, particularly in patients with liver dysfunction, limited its use [26].

Iron-based contrast agents, like Ferumoxytol, which was primarily used for iron deficiency anemia, has found off-label application in MRI, particularly for vascular imaging [25,27].

However, all of these “alternative contrast agents” have not achieved the widespread clinical use of gadolinium-based agents.

The first commercially available contrast agent incorporating gadolinium, [Gd(H_2_O)(dtpa)]^2−^ (Magnevist^®^), was synthesized in 1981 and approved by the Food And Drug Administration (FDA) for clinical use in 1988 [28].

While the FDA has approved several GBCAs, the precise number requires up-to-date information from official FDA resources. The EMA has taken a more cautious approach, especially regarding linear GBCAs, suspending the authorization of some due to gadolinium deposition concerns. The EMA approved macrocyclic GBCAs for breast MRI (dose of 0.1 mmol/kg) and recommended the suspension of certain linear GBCAs (Gadobenic acid, Gadodiamide, Gadopentetic acid, Gadoversetamide) due to concerns regarding gadolinium deposition in brain tissues and other organs [2,29].

Both the EMA and FDA have indeed approved Gadopiclenol, a novel non-ionic macrocyclic GBCA, introduced in 2022 [28]. Gadopiclenol is a very promising GBCA, which, in vitro, offers important advantages over existing macrocyclic agents thanks to its high stability (a longer dissociation half-life of 20 days vs. 18 h under acidic conditions) and twice-as-high relaxivity (11.6 mM^−1^ · sec^−1^ vs. 5 mM^−1^ · sec^−1^) [30].

Gadopentetate dimeglumine (Magnevist^®^), gadobenate dimeglumine (MultiHance^®^), gadoterate meglumine or gadoteric acid (Dotarem^®^), and gadobutrol (Gadovist^®^) are among the mostly used and explored GBCAs for breast MRI [31,32,33,34,35].

Gadopentetate dimeglumine (Magnevist^®^) has a gadolinium complex with diethylenetriaminepentaacetic acid (DTPA), salified with two molecules of N methylglucamine, in short Gd–DTPA–dimeg. This complex was the first compound approved for human administration, and was initially used to study central nervous system and spine lesions.

The introduction of polyaminopolycarboxylic ligands led to the development of other GCBAs, such as gadoterate meglumine (Gd–DOTA–meg, Dotarem^®^), formed by the gadolinium complex and the cyclic ligand DOTA (i.e., 1,4,7,10-tetraazacyclododecane-1,4,7,10-tetraacetic acid), or gadobutrol (Gadovist^®^), characterized by the presence of a cyclic ligand formally derived from DOTA, in which one of the acetic side arms has been replaced by a coordinating 2,3,4-trihydroxybutyl residue.

The peculiarity of gadobutrol is its formulation at a two-fold higher concentration of 1.0 M compared to most of the other GBCAs, formulated at a concentration of 0.5M. This implies that an equivalent volume of the gadobutrol formulation contains twice the number of GBCA molecules and that therefore the volume of gadobutrol necessary to achieve an approved dose is half that of the other available GBCAs.

Unlike the above-cited contrast agents that are extracellular and extravascular, gadobenate dimeglumine (Gd–BOPTA–dimeg, MultiHance^®^) is a targeted agent derived from modifications to the chemical structure of DTPA; it has a lipophilic residue, the benzyloxymethyl group, attached to the DTPA backbone, that allows transient albumin binding. This pharmacokinetic characteristic does make it suitable as either an unspecific extracellular agent for angiographic applications, or as a liver-specific agent [36].

Moreover, thanks to its weak and transient interaction with serum albumin, gadobenate dimeglumine has higher r1-relaxivity (6.7–7.9 L/mmol s^−1^ at 1.5 T) [30] compared to the other GBCAs used for breast MRI (gadopentetate dimeglumine, gadoterate meglumine, gadobutrol), ranging from 3.6 to 5.3 L/mmol s^−1^ at 1.5 T [37].

Higher r1-relaxivity translates into greater SI enhancement that, in turn, influences the diagnostic performance for detection and characterization of breast lesions. Different studies, indeed, demonstrated the superiority of gadobenate dimeglumine compared to that of gadopentetate dimeglumine at an equivalent dose. In particular, the study performed by Martincich et al. [31] showed a significantly greater breast cancer detection for all the three readers involved in the study and a significantly superior diagnostic performance in terms of sensitivity and accuracy for breast cancer detection with gadobenate dimeglumine relative to that achieved with gadopentetate dimeglumine at the same dose of 0.1 mmol/kg. This multicenter study confirmed the results highlighted by previously performed single-center, intraindividual crossover studies performed by Pediconi et al. [32,33].

A further confirmation of these results came from the study performed by Gilbert et al. [34], which compared gadobenate dimeglumine-enhanced MRI with gadopentetate dimeglumine-enhanced MRI, mammography, and ultrasound for breast cancer detection across different malignant lesion types and across different densities of breast tissue. Interestingly, a significantly improved cancer detection on MRI was noted in heterogeneously dense breasts (91.2–97.3% on gadobenate dimeglumine-enhanced MRI vs. 77.2–84.9% on gadopentetate dimeglumine-enhanced MRI vs. 71.9–84.9% with conventional techniques) and for invasive cancers (93.2–96.2% for invasive ductal carcinoma [IDC] on gadobenate dimeglumine-enhanced MRI vs. 79.7–88.5% on gadopentetate dimeglumine-enhanced MRI vs. 77.0–84.4% with conventional techniques). 

In addition to gadobenate dimeglumine and gadopentetate dimeglumine, other GBCAs have been explored as well. In particular, Pediconi et al. [38] showed the non-inferiority of gadobutrol compared with gadobenate for breast lesion detection (82.33% and 81.60%, respectively) and sensitivity (92.63% and 90.53%, respectively) in lesion characterization in breast MRI, at the equivalent doses of 0.1 mmol/kg body weight. A similar diagnostic performance of gadoteric acid and gadobutrol was demonstrated by Renz et al. [39]. However, this study highlighted a difference in some dynamic and morphologic breast lesion characterizations, with the initial signal increase being significantly higher for gadobutrol than for gadoteric acid for the malignant lesions.

Another study performed by Clauser et al. [35] compared a three-quarter (0.075 mmol/kg) dose of gadobenate dimeglumine, with a 1.5-fold higher dose than the on-label dose (0.15 mmol/kg) of gadoterate meglumine for breast lesion detection and characterization at 3T CE-MRI and demonstrated that gadobenate (84.5–88.7%) was not inferior to gadoterate (84.5–90.8%) in terms of lesion detection. In per-region analysis, gadobenate demonstrated higher specificity and accuracy compared with gadoterate. Multivariate analysis demonstrated superior, reader-independent diagnostic accuracy with gadobenate.

These results might be explained by the fact that gadobenate dimeglumine has the highest available r1-relaxivity while gadoterate meglumine has the lowest.

Despite these concerns, current evidence has not demonstrated any detrimental effects associated with gadolinium retention. Furthermore, the benefits of CE-MRI in these high-risk populations, particularly in early detection and management, outweigh the theoretical risks of gadolinium deposition.

### Adverse Effects of GBCAs

GBCAs are generally considered safe; however, they can occasionally cause adverse effects, from mild to severe, although severe reactions are rare [26] [Table 3].

Acute adverse events occur within one hour of contrast agent injection. The risk of occurrence is generally low; in particular, mild reactions are observed in 0.12% of patients, moderate reactions in 0.21%, and sever reactions in 0.03% [26]:

- Mild reactions: These are the most frequently reported adverse effects, including nausea, headache, vomiting, erythema, site injection reactions (discomfort, sensation of warmth or cold), and a metallic taste in the mouth [40].
- Moderate reactions: Hives, itching, facial swelling, dizziness, blood pressure variations, and irregular heart rhythms; medical intervention may be necessary [40].
- Severe reactions: These are exceedingly rare but can be life-threatening. These may involve severe allergic reactions (anaphylaxis), cardiac issues, or kidney injury, particularly in renal failure patients [40].

While less common, GBCAs can also cause other adverse reactions:

- Renal effects: GBCAs may occasionally exacerbate pre-existing renal problems, in addition to the risk of NSF, particularly linear GBCAs [41].
- Although rare, PC-AKI can occur after GBCA administration, especially in those with pre-existing kidney issues. The risk is considered minimal at standard doses for patients with eGFR ≥ 30 mL/min/1.73 m^2^.
- Gadolinium retention: Gadolinium deposition has been observed in various tissues such as the brain, bones, and skin, even in patients with normal kidney function [42,43].

Patients with renal failure face higher risk of this adverse reaction, although it has also been found in normal kidney function patients, and studies have shown greater correlation with linear GBCAs compared to macrocyclic ones [44].

According to the SIRM guidelines (2020) [45], PC-AKI (post-contrast acute kidney injury) risk after GBCA administration is minimal when the dosage is 0.1 mmol/kg. Currently, the use of gadolinium chelates cannot be considered a risk factor for PC-AKI in patients, with eGFR ≥ 30 mL/min/1.73 m^2^ [46].

Gadolinium retention refers to the deposition of gadolinium in the body after GBCA administration [44]. It has been observed in various tissues, including the brain, bones, skin, and kidneys [44,45,46,47].

Clinical symptoms potentially associated with gadolinium retention include arthralgia, muscle weakness, burning sensations, paresthesia, altered temperature perception, fever, flu-like symptoms, fatigue, nausea, vomiting, headache, dizziness, brain fog, and visual disturbances [48]. In some cases, patients may also develop cutaneous nodules on distal extremities, known as gadolinium-associated plaques (GAPs) [49]. Kanda et al. (2014) were the first to document increased signal intensity in the dentate nucleus and globus pallidus on unenhanced T1-weighted images, attributing these findings to prior GBCA administrations [50,51]. However, in the specific context of breast MRI, Neal et al. [52] found no evidence of gadolinium retention in the deep brain nuclei of women who underwent breast MRI with gadoteridol. Nevertheless, future research is warranted to elucidate any potential links and long-term effects of gadolinium exposure, especially in high-risk individuals undergoing annual CE-MRI as part of a screening protocol [52]. Additionally, some less stable GBCAs may induce nephrogenic systemic fibrosis (NSF), particularly in patients with renal failure. NSF is a serious, delayed adverse reaction characterized by fibrotic thickening of the extremities and, in some cases, the trunk [53]. NSF should only be diagnosed when the clinical and histopathological criteria outlined by the YALE NSF Registry are met [54]. In 2006, NSF has been associated with the administration of certain GDCAs in RI patients [55].

Higher-risk patients are those with GFR < 15 mL/min/1.73 m^2^, those on hemodialysis or peritoneal dialysis, and patients with reduced renal function who have had or are awaiting liver transplantation [53]. Although the precise pathogenesis of NSF remains unclear, prevailing hypotheses suggest that prolonged retention of gadolinium may lead to dissociation of the gadolinium ion from it chelates. This dissociation results in free gadolinium, which is believed to play a critical role in the development of fibrosis [56]. To minimize the risk of NSF, serum creatinine and eGFR are required as screening for renal function before administering GBCAs and, as a rule, GBCA administration is contraindicated in patients with eGFR below 15 mL/min/1.73 m^2^ [57].

## 4. Future Perspectives

### 4.1. Emerging Contrast Agents

The introduction of high-relaxivity GBCAs addresses this need by enabling lower doses of gadolinium to achieve comparable or superior contrast enhancement. These agents are expected to improve diagnostic accuracy and patient management, while also reducing potential risks associated with gadolinium deposition [58,59]. High-relaxivity GBCAs offer several benefits, including enhanced contrast at standard doses or equivalent contrast using reduced gadolinium doses [60].

Recent advancements have led to the approval of gadopiclenol (Elucirem™), a novel macrocyclic, high-relaxivity GBCA. Approved by the FDA in September 2022 and by the European Medicines Agency (EMA) in December 2023 [61], gadopiclenol is indicated for detecting and visualizing lesions with abnormal vascularity in the central nervous system (CNS) and various other organs, including the head and neck, thorax, abdomen, pelvis, breast, and musculoskeletal system, at a dose of 0.05 mmol/kg [30].

The relaxation rate R1, which reflects a contrast agent’s effectiveness, is influenced by the interaction of water molecules with the gadolinium (Gd^3+^) ion. This includes the inner sphere contribution (directly bound water molecules) and the outer sphere contribution (nearby diffusing water molecules) [30].

Relaxivity can be enhanced by modifying GBCAs’ molecular properties, such as increasing molecular size to slow rotational motion or increasing the hydration number, which represents the number of water molecules that can directly interact with the Gd^3+^ ion [62].

Most current-generation GBCAs have a hydration number of q = 1 q, whereas gadopiclenol achieves q = 2 q, doubling the number of water molecules involved in proton exchange and resulting in significantly higher relaxivity [30].

Gadopiclenol exhibits the highest r1-relaxivity among non-specific GBCAs, with values of 12.8 and 11.6 mM/s at 1.5 T and 3.0 T, respectively, in water and human serum at 37 °C [30].

This represents a 2- to 3-fold improvement over current agents, without evidence of protein binding. Its high thermodynamic stability and kinetic inertness further contribute to its favorable in vivo behavior. At pH 1.2, gadopiclenol demonstrates a dissociation half-life of 20 ± 3 days, approximately four times greater than the current generation of GBCAs. Additionally, its low osmolality and high solubility enable administration of low doses and small volumes without compromising diagnostic performance [30].

Preclinical and clinical studies have shown that gadopiclenol achieves equivalent diagnostic efficacy at 0.05 mmol/kg, half the standard dose of current GBCAs like gadobutrol [62,63,64,65].

Two phase III trials demonstrated non-inferiority of gadopiclenol in CNS and body MRI settings, confirming its ability to provide comparable lesion visualization at reduced gadolinium doses [62,63].

A randomized, double-blind, crossover, phase III study conducted between August 2019 and December 2020 across 33 centers in 11 countries evaluated gadopiclenol against gadobutrol [63]. Participants included adults with at least one suspected focal lesion in one of three body regions: the head and neck; breast, thorax, abdomen, or pelvis; or musculoskeletal system. Each participant underwent two contrast-enhanced MRI examinations, randomized to receive gadopiclenol or gadobutrol first. Among the 273 participants (mean age, 57 ± 13 years; 162 women) who underwent both MRI examinations, 260 without major protocol deviations were included in the non-inferiority analysis. Gadopiclenol was found to be non-inferior to gadobutrol for all qualitative visualization parameters, with the lower limit of the 95% confidence interval for differences being above the pre-defined non-inferiority margin of −0.35. Between 75% and 83% of participants had no clear preference for images enhanced with either contrast agent. These findings demonstrate that gadopiclenol at 0.05 mmol/kg is as effective as gadobutrol at 0.1 mmol/kg for lesion evaluation in contrast-enhanced body MRI and exhibits a similarly favorable safety profile.

Safety data from these studies and post-marketing surveillance indicate that gadopiclenol has a favorable safety profile, with no serious adverse events reported after over 882,550 administrations in the United States and a very low rate of non-serious adverse events (1 case per 27,580 exposures) [30].

In addition to gadopiclenol, other agents are under development to further improve relaxivity. Gadoquatrane (BAY 1747846) is a tetrameric, macrocyclic, extracellular GBCA currently in late-stage clinical development. This agent employs a multimeric system with four macrocyclic Gd-GlyMe-DOTA cages per molecule, resulting in a molecular weight of 2579 g/mol and reduced rotational motion [64].

Gadoquatrane exhibits an r1r_1r1-relaxivity of 11.8 and 10.5 mM/s at 1.5 T and 3.0 T, respectively, in human plasma at 37 °C and pH 7.4, which is significantly higher than most marketed GBCAs, and preclinical data have demonstrated that gadoquatrane provides a superior contrast-to-noise ratio compared to gadobutrol at equivalent doses in MRI studies of rat glioblastoma models [64]. A first-in-human study assessing pharmacokinetics and safety showed no serious adverse events across dose levels of 0.025 to 0.2 mmol/kg, with a favorable safety profile and no evidence of QT/QTc prolongation at clinical doses [65].

Finally, alternative agents based on other metals have been explored for their potential to replace GBCAs, providing effective imaging while reducing associated risks.

At present, new Mn-based agents are currently under investigation in preclinical and early clinical studies, showing potential as safer and more effective alternatives [66,67].

Ultrasmall superparamagnetic particles of iron oxide (USPIOs) represent another promising alternative for MR imaging. Iron has long been recognized as a viable alternative to gadolinium for MR contrast media due to its paramagnetic properties. Iron oxide shortens T2 relaxation time, significantly reducing MR signal intensity and generating a negative contrast effect [68].

In particular, USPIOs were studied in experimental applications for visualizing lymph node (LN) metastases [27]. This method offers the advantage of avoiding ionizing radiation while enabling the detection of small metastatic nodes. USPIOs are naturally taken up by macrophages and accumulate in normal lymph nodes, creating areas of low signal intensity. Regions within the node that retain a higher signal are likely involved with tumors, although fibrosis or inflammation can also account for this finding.

Despite its potential, the lack of approved and clinically available USPIOs limits widespread adoption and larger-scale clinical studies. Proposed applications for USPIO MRI, along with specific compounds and imaging protocols, remain an area of active research [27].

### 4.2. Abbreviated Breast MRI

Breast MRI presents some limits including high direct and indirect costs, unequal accessibility worldwide, and higher patient discomfort [69,70]. In particular, a prominent obstacle is the considerable cost related to MRI technology and contrast agents and the time spent in conducting an MRI examination, hampering its accessibility and widespread use in the world [69,70,71]. The choice of using an abbreviated protocol, incorporating kinetic analysis information, may help to mitigate these barriers [72]. For this reason, in 2014 Kuhl et al. introduced, for the first time, the concept of an abbreviated protocol in breast MRI (AB-MRI) [73].

The rationale behind the development of abbreviated breast MRI (AB-MRI) protocols is the omission of non-critical sequences to reduce examination duration, enhance patient comfort, and increase imaging throughput, while simultaneously decreasing interpretation time and associated costs [73]. Consequently, numerous studies have explored and proposed various versions of AB-MRI protocols. However, as this remains a relatively novel approach, key issues are yet to be fully defined—particularly concerning which specific sequences should be included or excluded. Currently, there is no consensus on the optimal protocol configuration to ensure maximal diagnostic performance.

Many of the recently published studies [74,75,76] have proposed an AB-MRI protocol based on a pre-contrast and single first post-contrast T1-weighted image, with or without the addition of further unenhanced sequences. The use of only the first post-contrast T1WI is based on the evidence that tumors, having an extensive neo-angiogenesis, typically present early contrast enhancement. Of course, the lack of kinetic information and more-delayed enhanced sequences might reduce the sensitivity [76] of the abbreviated protocol, and this is especially true for slowly enhancing malignancies such as low-grade ductal carcinomas in situ or invasive lobular carcinomas; these might be overlooked by AB-MRI, which lacks delayed post-contrast phases, leading to missed diagnoses.

The other face of the coin is that late-enhancing benign lesions account for a high rate of false positives [74], requiring further investigations, increasing costs and the patient’s anxiety.

A possible way to address these challenges, while still preserving AB-MRI’s advantages, is a protocol based on ultra-fast contrast-enhanced MRI. This is a new imaging technique that demonstrates the early wash-in of contrast agents with high temporal resolution while preserving necessary diagnostic spatial resolution [77].

Recently, Ying Cao et al. using an ultrafast DCE-MRI protocol, correctly identified about 70.91% of unnecessary biopsies that could potentially be avoided [78].

More research, possibly using large-scale and multicenter prospective studies, is needed to further validate AB-MRI’s diagnostic accuracy and ascertain its cost-effectiveness, acceptability, and practicality in both diagnostic and screening contexts.

### 4.3. Role of MRI in Breast Evaluation in Plastic Surgery

MRI plays a role in breast evaluation for plastic surgery, offering information for both reconstructive and aesthetic procedures, and providing detailed insights into breast tissue composition, including fat, glandular tissue, blood vessels, and lymph nodes [16,79].

In reconstructive surgery, MRI excels at evaluating breast implant integrity and detecting ruptures or leaks [16,79,80]. It is also increasingly important for assessing fat graft survival and volume retention after fat transfer procedures [79].

It allows for quantitative volumetric analysis, helping determine how much of the grafted tissue survives and integrates into the breast tissue, allowing surgeons to refine their techniques and provide more accurate predictions to patients [79,80].

## 5. Conclusions

Technological advancements, including multiparametric and abbreviated protocols and high-relaxivity GBCAs, continue to refine their diagnostic capabilities while addressing safety concerns. Recent innovations, such as gadopiclenol, demonstrate promising potential to reduce gadolinium exposure and enhance diagnostic performance.

Future efforts should focus on bridging the gap between advanced diagnostic tools and global healthcare disparities, ensuring that all patients can benefit from the life-saving potential of breast MRI.

## Figures and Tables

**Table 2:** Gadolinium-based contrast agents (GBCAs) [24].

Structure	Ionic/Non-Ionic	Contrast-Agents (Commercial Name)
Linear	Ionic	Gadopentetate (Magnevist)
Linear	Non-ionic	Gadodiamide (Omniscan)
Linear	Non-ionic	Gadoversetamide (Optimark)
Macrocyclic	Ionic	Gadoterate (Dotarem)
Macrocyclic	Non-ionic	Gadobutrol (Gadovist)
Macrocyclic	Non-ionic	Gadoteridol (ProHance)

**Table 3:** Contrast agents’ acute adverse reactions [33].

	Hypersensitivity/Allergy-like	Chemotoxic
Mild	Mild urticaria Mild itching Erythema	Nause/mild vomiting Warmth/chills Anxiety Vasovagal-reaction (spontaneously resolved)
Moderate	Marked urticaria Mild bronchospasm Facial/laryngeal edema	Vasovagal-reaction
Severe	Hypotensive shock Respiratory attest Cardiac arrest	Arrythmia Convulsion
